# Hypertrophy of the Lingual Tonsil

**Published:** 1897-06

**Authors:** W. E. Adams

**Affiliations:** Augusta, Ga.


### HYPERTROPHY OF THE LINGUAL TONSIL.

BY W. E. ADAMS, M.D.,

AUGUSTA, GA.

A great deal has been written and published on the subject of hypertrophied lingual tonsils during the past year, and my excuse for adding to this mass of literature is to give in a condensed form the more salient points from the papers of others and my experience in the hospitals of New York and Philadelphia. The subject is fraught with much that is of vital importance to the general practitioner as well as the specialist, and I shall endeavor to show that failure to make correct diagnosis frequently results in an unfavorable termination of a simple and easily curable malady.

The fourth, or lingual, tonsil is situated at the base of the tongue in the glosso-epiglottic fossæ, and constitutes the lower portion of what is termed the lymphoid ring. It is about one-sixth of an inch in thickness and irregularly flat in shape.

That this lymphoid tissue is frequently the seat of inflammatory action is well established, and that chronic hypertrophy is frequently the ultimate result has been abundantly proven. Its situation and profuse vascularity would suggest numerous causes for inflammation. The passage of food over it may produce irritation, specific and infectious diseases, rheumatic and gouty diatheses, digestive derangements, any inflammatory condition of the nasal chambers, pharynx and larynx may involve it. With this array of causes its frequency is not surprising. Its excessive vascularity and somewhat dependent position has a tendency to aggravate the inflammatory process and result in permanent enlargement. I cannot agree with some writers that hypertrophy of the lingual tonsil is confined to adult life. This tissue is analogous to the pharyngeal tonsil, and lymphoid changes belong essentially to childhood. Hypertrophy of the lingual tonsil may occur at any age, but does not manifest itself until of sufficient size to produce irritative symptoms by impinging upon the surrounding parts.

The symptoms are as numerous as the causes. Irritating, hacking cough with little or no expectoration, occasionally slight hemorrhage from rupture of varicose veins, sometimes temperature ranges from 100 to 104 degrees, sensation of foreign body in throat not removed by deglutition, constant endeavor to clear throat with no relief, feeling of stiffness and sometimes pain, nausea and vomiting, laryngeal fatigue and inability to use voice with comfort and ease, occasionally sharp pain in ear.

The hacking cough, hemorrhage, elevation of temperature, mental worry and consequent emaciation may cause both physician and patient to suspect phthisis. Cough mixtures directed to the pulmonary and bronchial tissues are frequently prescribed, and the failure to relieve or benefit the condition, to all appearances, establishes the diagnosis of the dreadful malady. It may be justly claimed that this error in diagnosis should not occur.

The sensation of "foreign body or lump in the throat" not removed by deglutition occurring in a female of nervous temperament easily misleads to the diagnosis of hysteria with the characteristic globus hystericus. The usual treatment for hysteria would of course be ineffectual, and the chances of producing the real article would be enhanced by failure to relieve the symptoms.

Nausea and vomiting, sometimes produced by hypertrophy of the lingual tonsil, directs attention to the alimentary canal, and here, too, drugs given to allay the alimentary irritation would be irrational and inefficacious. And so I might mention other groups of symptoms produced by hypertrophy of the lingual tonsil which would be characteristic of some malady differing in every detail from the real condition.

The diagnosis is easily made by careful examination with the laryngoscope. In all cases, with persistence of any or all of the above mentioned symptoms, careful examination of the upper air passages for reflex causes should not be neglected.

The treatment is simple. Attention to the nasal chambers and pharynx, the application of astringents, as nitrate of silver in two to ten per cent. solution, chloride of zinc two to four per cent. solution, tincture iodine, tannic acid and glycerin, chromic acid, nitric acid, removal by cold wire snare. As a rule, application of astringents should be made every second day. If necessary, tonics should be administered. Complete relief is obtained in all cases if treatment is thorough and persistent.

*810 Broadway.*

